# Enhancing Precision in Rectal Cancer Care: Unravelling the Correlation Between MRI Locoregional Staging and Prognostic Factors, With Post-resection Pathology

**DOI:** 10.7759/cureus.74592

**Published:** 2024-11-27

**Authors:** Manoj S Gowda, Bennett C Peter, Mesut E Enc, Sundaram V Gowtham, Sri Vishnu Thulasiraman, Mohamed Mansour, Trisha Jha, Madan Jha

**Affiliations:** 1 General Surgery, Royal Victoria Infirmary, Newcastle, GBR; 2 General Surgery, James Cook University Hospital, Middlesbrough, GBR; 3 Trauma and Orthopaedics, Royal Victoria Infirmary, Newcastle, GBR; 4 General Surgery, Kovai Medical Center and Hospital, Coimbatore, IND; 5 Surgery, James Cook University Hospital, Middlesbrough, GBR; 6 Surgery, Keele University School of Medicine, Newcastle, GBR

**Keywords:** colonoscopy, colorectal cancer, computed tomography, magnetic resonance imaging, mesorectum, mri pelvis, oncological outcomes, preoperative assessment, prognostic tool, rectal cancer

## Abstract

Introduction

Magnetic resonance imaging (MRI) serves as a pivotal tool in the preoperative assessment of rectal cancer. This study aims to evaluate the accuracy of preoperative MRI pelvis in rectal cancer for locoregional staging, circumferential margin (CRM+), and vascular invasion (V1) with postoperative histopathological findings.

Methods

All patients who underwent preoperative staging MRI pelvis scanning for histology-proven primary rectal adenocarcinoma between January 2020 and July 2022 were included in this study. Preoperative MRI assessment data, including tumor (T), nodal (N) staging, and other prognostic factors, including circumferential margin and vascular invasion, were compared with histopathological reports. Sensitivity, specificity, negative predictive value (NPV), positive predictive value (PPV), and accuracy were calculated. Agreement between MRI and pathology reports was evaluated using the weighted kappa statistic.

Results

The study included 143 patients. The accuracy of MRI is at its peak for T0 and T4 tumors (T0-97.9%, T4-93.37). The weighted kappa statistic for T staging is 0.401, and for N staging, it is 0.286. The standard kappa statistic for extramural vascular invasion (V) and circumferential resection margin (CRM) is 0.269 and 0.225, respectively.

Conclusion

MRI demonstrates fair agreement with pathology regarding T and N staging and exhibits high specificity for CRM and V1.

## Introduction

According to the GLOBOCAN database, gastrointestinal (GI) cancers are responsible for a significant global health burden, accounting for approximately 25% of all newly diagnosed cancer cases and contributing to around 33% of cancer-related deaths worldwide. Among the various types of GI cancers, rectal cancer alone represents 3.8% of newly diagnosed cancer cases and is responsible for 3.4% of all cancer-related mortality [1]. These statistics highlight the importance of early detection, accurate staging, and appropriate treatment for rectal cancer to improve patient outcomes and reduce its mortality rate.

The prognosis of rectal cancer is influenced by a variety of factors. Key determinants include the tumor’s extent, the involvement of regional lymph nodes (N+), the tumor’s histological grade, circumferential resection margin (CRM) involvement, and the presence of extramural vascular invasion (V0/V1) [2]. These factors are critical in determining the appropriate course of treatment for patients diagnosed with rectal cancer, as they provide important information about the tumor’s aggressiveness and the likelihood of disease spread.

A multidisciplinary team (MDT) approach is now considered the gold standard for managing rectal cancer, and research has shown that this collaborative approach leads to improved patient outcomes [3]. The MDT is composed of various specialists, including oncologists, radiologists, surgeons, and pathologists, who work together to assess the patient’s condition from different medical perspectives. They consider the aforementioned prognostic factors - tumor size, lymph node involvement, and CRM status, among others - when planning the patient's treatment regimen. This practice helps ensure that the patient receives a tailored treatment plan that minimizes the risk of both under-treatment and overtreatment, which can significantly impact patient prognosis and quality of life.

One of the essential tools for preoperative assessment in rectal cancer is magnetic resonance imaging (MRI) of the pelvis. MRI is particularly valuable because it provides high-resolution images that can reveal critical details about the tumor’s local extent, including its proximity to surrounding tissues, involvement of the circumferential resection margin (CRM), and signs of vascular invasion. MRI is therefore widely used by the MDT as part of the preoperative staging process to accurately determine the stage of the disease and to assist in formulating a comprehensive treatment plan. By assessing these key factors, MRI helps clinicians decide whether surgery, chemotherapy, radiation therapy, or a combination of treatments is most appropriate for the patient.

The focus of this study is to evaluate the accuracy of MRI in the locoregional staging of rectal cancer. Specifically, the study assesses how well MRI can identify CRM involvement and vascular invasion, which are crucial for determining the likelihood of complete tumor resection and the potential for metastasis.

This article was originally presented as a poster at the Association of Laparoscopic Surgeons of Great Britain and Ireland (ALSGBI) Conference held in Portsmouth on November 31, 2023. The findings from this research aim to contribute to the growing body of evidence supporting the use of MRI in the accurate preoperative assessment and staging of rectal cancer, ultimately improving patient outcomes by guiding more precise treatment planning.

## Materials and methods

For this study, we analyzed data from a prospectively maintained register of patients diagnosed with colorectal cancers, eliminating the need for ethics approval. All patients included in the study had undergone a staging MRI scan and were confirmed to have primary histology-proven rectal adenocarcinoma. The study spanned from January 2020 to July 2022 and was conducted at a single tertiary colorectal cancer center in the United Kingdom. Rectal cancer, for the purposes of this study, was defined as an adenocarcinoma located within 15 cm of the anal verge, as determined by endoscopy, with the proximal extent of the tumor positioned at or below the sacral promontory on MRI imaging.

The dataset used for this study provided comprehensive information, including the patient's age, gender, preoperative endoscopy results, histology findings, MRI scan details, outcomes from multidisciplinary team (MDT) meetings, and final histopathology reports from the resected specimens. The inclusion criteria were patients who underwent a preoperative MRI for rectal cancer staging, whereas those with other types of colorectal cancer or those who were only candidates for best supportive care or palliative treatment were excluded from this analysis.

The role of MRI in this study was pivotal, as preoperative MRI scans were systematically reviewed and discussed in MDT meetings. The MDT included various healthcare professionals: colorectal surgeons, gastroenterologists, oncologists, radiologists, pathologists, and colorectal nurse practitioners, all contributing to comprehensive case discussions. MRI reports provided critical data such as the extent of the primary tumor, lymph node involvement, vascular invasion status (classified as V0 or V1), circumferential resection margin (CRM) involvement, the craniocaudal extent of the tumor, and the distance from the anal verge. These reports were available through online MDT records, ensuring consistent review and decision-making regarding patient care.

Following surgery, the final pathology reports from the resected specimens were similarly reviewed and discussed within the MDT to determine the next steps in each patient's management plan. Any deviations between preoperative MRI findings and final pathology were also thoroughly evaluated. The exclusion criteria for this study involved patients who were not considered for surgery, either due to being offered palliative treatment or having other colorectal cancer types apart from rectal cancer.

MRI imaging was performed using either a Siemens Magnetom Skyra 3T (Siemens, Munich, Germany) or a Siemens Magnetom Avanto 1.5T machine, utilizing both coronal and axial T1- and T2-weighted views. A standardized set of parameters was employed across all MRI scans to ensure consistency in the staging process. These parameters included a repetition time (TR) of 5650 ms, an echo time (TE) of 88 ms, a field of view (FOV) of 200 mm, and a slice thickness of 3 mm. The average time for each MRI session was approximately 25 minutes, providing detailed images necessary for staging.

In terms of statistical analysis, continuous variables, such as age and the number of lymph nodes removed or involved, were expressed as mean, median, range, and standard deviation values. The accuracy of MRI in identifying key pathological features, such as tumor extent, lymph node involvement, and vascular invasion, was assessed by calculating sensitivity, specificity, positive predictive value (PPV), negative predictive value (NPV), and overall accuracy. These metrics were calculated based on true positive, true negative, false positive, and false negative values derived from the comparison between MRI findings and final pathology reports.

To evaluate the level of agreement between MRI staging and pathology reports, Kappa statistics were used. The Kappa scale provides a measure of concordance, where values less than 0 indicate no agreement, 0 to 0.20 indicate slight agreement, 0.21 to 0.40 indicate fair agreement, and 0.41 to 0.60 indicate moderate agreement. In this study, statistical significance was defined as p < 0.05, indicating that any findings with a p-value below this threshold were considered statistically significant.

All data were collected and analyzed using IBM SPSS software, version 29 (IBM Corp, Armonk, NY, US). This software allowed for detailed statistical evaluation and ensured that the results were both accurate and reliable. The study's findings contribute to understanding the role of MRI in preoperative staging, helping ensure that rectal cancer patients receive the most appropriate and effective treatment based on accurate staging information.

## Results

Based on the inclusion and exclusion criteria set for the study, a total of 143 patients were ultimately included. These patients had a median age of 71 years, with an age range spanning from 29 to 92 years. The male-to-female ratio of participants was 1.86:1, indicating a higher proportion of males compared to females. The demographic details, as well as the pathological characteristics of the patients, are summarized in Table 1.

**Table 1:** Demographics and pathological characteristics T-tumor stage, N-nodal stage, V-extramural vascular invasion (0-not involved, 1-involved), CRM-circumferential resection margin (0-not involved, 1-involved) p<0.05 is considered significant

Characteristics			
Age	Value		
Mean	68.52		
Median	71		
Range	29-92		
Standard deviation	11.59		
Sex			
Male	93		
Female	50		
T-stage	MRI	Pathology	P-value (chi-square test)
T0	3	2	0.18
T1	19	34	
T2	46	35	
T3	69	65	
T4	6	7	
N stage			
N0	100	98	0.83
N1	31	30	
N2	12	15	
Extramural vascular invasion			
V0	129	89	<0.0001
V1	14	54	
Circumferential resection margin			
CRM0	127	137	0.02
CRM1	16	6	

The collected data reveal that the accuracy of MRI in determining tumor stage (T stage) was highest for both T0 and T4 tumors. Specifically, MRI accuracy for T0 tumors was 97.9%, while for T4 tumors, it was 93.37%. These numbers demonstrate that MRI is particularly reliable at both ends of the tumor staging spectrum, meaning that it performs well in identifying either very early-stage tumors or tumors that have significantly progressed. However, MRI accuracy for intermediate stages of rectal cancer was notably lower. For T1 tumors, MRI had an accuracy rate of 77.57% while T2 tumors showed an accuracy rate of 62.94%. The accuracy for T3 tumors was only slightly higher than T2 at 66.92%, as outlined in Table 2.

**Table 2:** Sensitivity, specificity, PPV, NPV, PLR, NLR, and accuracy of the MRI rectum PPV-positive predictive value, NPV-negative predictive value, PLR-positive likelihood ratio, NLR-negative likelihood ratio, T-tumor stage, N-nodal stage, V-extramural vascular invasion (0-not involved, 1-involved), CRM-circumferential resection margin (0-not involved, 1-involved)

MRI	T0	T1	T2	T3	T4	N0	N1	N2	V0/V1	CRM0/1
Sensitivity [n (%)]	100	38.24	40	66.15	28.57	51.11	26.67	26.67	24.07	50
Specificity [n (%)]	97.87	94.5	70.37	67.95	97.06	79.6	79.65	93.75	98.88	90.51
PPV [n (%)]	40.02	74.93	30.43	73.65	35.58	91.4	32.13	96.54	93.83	24.63
NPV [n (%)]	100	78.05	78.36	59.72	95.98	27.72	75.04	16.37	64.71	96.69
PLR	47	6.96	1.35	2.06	9.71	1.63	2.5	4.27	21.43	5.27
NLR	0	0.65	0.85	0.5	0.74	0.4	0.61	0.78	0.77	0.55
Accuracy [n (%)]	97.9	77.57	62.94	66.92	93.37	75.81	65.59	35.58	67.81	88.15

The accuracy of MRI in determining nodal involvement (N staging) appeared to decline as the nodal stage increased. For patients with no lymph node involvement (N0), the MRI accuracy was 75.81%. However, this accuracy dropped to 65.59% for the N1 stage, and it further decreased to 35.58% for the N2 stage, indicating that MRI becomes less reliable in identifying more advanced nodal disease.

Regarding the circumferential resection margin (CRM) involvement, MRI had a sensitivity of 50% and a high specificity of 90.51%. This means that while MRI was less effective at correctly identifying all true positive cases of CRM involvement (sensitivity), it was highly effective at correctly identifying true negative cases (specificity). The overall accuracy of MRI in detecting CRM status was calculated at 88.15%, demonstrating that MRI is quite reliable for assessing CRM involvement in most cases.

The weighted kappa statistic, which measures the level of agreement between MRI staging and the final pathological findings, was 0.401 for T staging and 0.286 for N staging. This suggests moderate agreement for T staging and fair agreement for N staging. The standard kappa statistic was also calculated for two other important prognostic factors: extramural vascular invasion (V) and circumferential resection margin (CRM) involvement. The kappa value for extramural vascular invasion was 0.269, with a 95% confidence interval (CI) ranging from 0.137 to 0.400. Similarly, the kappa value for CRM involvement was 0.225, with a 95% CI ranging from 0.021 to 0.471. Both values suggest a fair level of agreement between MRI and final pathology findings for these parameters.

In terms of sensitivity and specificity, MRI demonstrated varying levels of effectiveness in predicting different pathological features. For CRM involvement, MRI had a sensitivity of 50% and a specificity of 90.5%. For vascular invasion (V1), MRI had a sensitivity of 24.1% and an exceptionally high specificity of 98.9%, indicating its high reliability in ruling out false positives. For nodal involvement (N+), MRI demonstrated a sensitivity of 51% and a specificity of 79.6%, showing moderate effectiveness in identifying patients with lymph node involvement.

## Discussion

This study evaluated the diagnostic performance of MRI in the locoregional staging of rectal cancer, focusing on important prognostic factors such as tumor staging (T), nodal involvement (N), circumferential resection margin (CRM), and extramural vascular invasion (V1). The weighted kappa statistics for T and N staging in our study were 0.401 and 0.286, respectively, indicating fair agreement between MRI findings and postoperative pathology. These results align with those of other research, such as a study by Liping Xu et al. [4], which included 354 patients and reported kappa values of 0.625 and 0.323 for T and N staging, respectively. Similarly, a study by Zhou et al., which focused on nodal staging in rectal cancer, showed a kappa value of 0.348 for MRI in preoperative nodal staging, demonstrating fair agreement with histopathology. This allowed for more targeted and precise therapy in these patients [5].

In our study, the accuracy of MRI was highest for detecting T0 and T4 rectal tumors, with accuracy rates of 97.9% and 93.37%, respectively. Sensitivity was also highest for T0 and T3 stages, while specificity was most accurate for T0, T1, and T4 tumors. These findings are consistent with a large meta-analysis, which evaluated the diagnostic accuracy of MRI in rectal cancer staging across 35 studies. The meta-analysis reported the highest sensitivity for diagnosing T3 tumors at 80%, and the highest specificity for T1 and T4 tumors at 97% [6]. This suggests that MRI remains a reliable tool for both early and advanced tumor stages, although its accuracy can vary in intermediate stages.

Regarding nodal status (N staging), MRI accuracy in our study was highest for N0 (75.81%), followed by N1 (65.59%), but it was significantly lower for N2 (35.58%). The decreased accuracy for N2 is likely due to the difficulty in detecting smaller metastatic lymph nodes (<4 mm). A Chinese study comparing CT and MRI for lymph node evaluation found similar results, reporting 59.6% accuracy when lymph node borders were used as a criterion and 61.5% accuracy when lymph node enhancement was used [5]. Another study reported MRI accuracy for N staging at 56.8%, with N0, N1, and N2 accuracy rates of 49.8%, 43.7%, and 64.2%, respectively [4]. In comparison, our study shows MRI's accuracy is highest for N0, with a decline in accuracy as the nodal stage increases, emphasizing the challenges in detecting nodal involvement in advanced disease.

For CRM involvement, MRI demonstrated moderate sensitivity (50%) and high specificity (90.5%). These results are comparable to those reported in a meta-analysis by Al Sukhni et al. [7], where MRI showed a sensitivity of 77% and a specificity of 94%. Additionally, another meta-analysis of 9 studies, encompassing 529 patients, reported an overall specificity of 85% for detecting CRM involvement [8]. High specificity ensures that MRI is effective in ruling out false positives for CRM involvement, although the sensitivity needs improvement to accurately detect all positive cases.

In terms of vascular invasion (V1), MRI showed high specificity in our study (98.88%) but low sensitivity (24.07%), with an overall accuracy of 67.87%. These findings are consistent with a study by Sohn et al. [9], which included 447 patients and reported MRI sensitivity, specificity, and accuracy for detecting V1 at 28.2%, 94%, and 80.3%, respectively. The lower sensitivity in detecting vascular invasion is likely due to challenges in identifying small blood vessels (<3 mm) invaded by cancer, as well as limitations in spatial resolution and the presence of partial volume artifacts on MRI.

However, our study has several limitations. First, the patients were scanned using two different MRI machines, one with 1.5 Tesla resolution and the other with 3 Tesla resolution, which could lead to variations in image quality and diagnostic performance. Further research should explore whether the difference in scanner resolution contributes to the under-reporting of certain features. Second, this study was conducted at a single center, which limits the generalizability of the findings. Additional data from other centers are necessary to corroborate these results. Lastly, while multiple MRI specialists interpreted the scans, there is a potential for operator bias. Nonetheless, this bias is minimized as the scans are consistently reviewed by a dedicated gastrointestinal radiologist during multidisciplinary team (MDT) discussions, ensuring a more standardized approach to interpretation.

## Conclusions

Preoperative imaging in rectal cancer is vital in the staging of disease, leading to different treatment protocols for different patients. Our study was done to check the efficacy of MRI pelvis in preoperative staging of rectal cancers. MRI exhibits fair agreement with pathology regarding T and N staging and should be utilized preoperatively for rectal cancer staging. MRI demonstrates higher accuracy in T0 and T4 tumors than in other T stages. Moreover, MRI displays high specificity and low sensitivity for V1 and CRM. With the improvement of imaging modalities, further randomized controlled trials are needed to identify potential areas to improve the accuracy of preoperative staging.
